# A Targeted Complement Inhibitor CRIg/FH Protects Against Experimental Autoimmune Myasthenia Gravis in Rats *via* Immune Modulation

**DOI:** 10.3389/fimmu.2022.746068

**Published:** 2022-01-26

**Authors:** Jie Song, Rui Zhao, Chong Yan, Sushan Luo, Jianying Xi, Peipei Ding, Ling Li, Weiguo Hu, Chongbo Zhao

**Affiliations:** ^1^ Department of Neurology, Huashan Hospital Fudan University, Shanghai, China; ^2^ National Center for Neurological Disorders, Shanghai, China; ^3^ Fudan University Shanghai Cancer Center and Institutes of Biomedical Sciences, Shanghai Medical College, Fudan University, Shanghai, China; ^4^ Key Laboratory of Breast Cancer in Shanghai, Fudan University Shanghai Cancer Center, Fudan University, Shanghai, China

**Keywords:** myasthenia gravis, complement inhibitor, factor H, CRIg, cytokine, Treg

## Abstract

Antibody-induced complement activation may cause injury of the neuromuscular junction (NMJ) and is thus considered as a primary pathogenic factor in human myasthenia gravis (MG) and animal models of experimental autoimmune myasthenia gravis (EAMG). In this study, we tested whether CRIg/FH, a targeted complement inhibitor, could attenuate NMJ injury in rat MG models. We first demonstrated that CRIg/FH could inhibit complement-dependent cytotoxicity on human rhabdomyosarcoma TE671 cells induced by MG patient-derived IgG *in vitro*. Furthermore, we investigated the therapeutic effect of CRIg/FH in a passive and an active EAMG rodent model. In both models, administration of CRIg/FH could significantly reduce the complement-mediated end-plate damage and suppress the development of EAMG. In the active EAMG model, we also found that CRIg/FH treatment remarkably reduced the serum concentration of autoantibodies and of the cytokines including IFN-γ, IL-2, IL-6, and IL-17, and upregulated the percentage of Treg cells in the spleen, which was further verified *in vitro*. Therefore, our findings indicate that CRIg/FH may hold the potential for the treatment of MG *via* immune modulation.

## Introduction

The complement system is a major component of innate immunity and plays a crucial role in the pathogenesis of several inflammatory and autoimmune diseases (1). The complement cascade is composed of more than 30 soluble and membrane-bound proteins, which could be initiated through three pathways, i.e., classical pathway (CP), alternative pathway (AP), and lectin pathway (LP) (2). After activation, complement exerts its functions mainly through the membrane attack complex (MAC, C5b-9n), the opsonin molecules (C3b/iC3b/C3d), and the anaphylatoxins (C3a and C5a) (3). All cell-bound C3 convertases can induce the “amplification loop” of AP through their common product C3b, which contributes to 80%–90% of total complement activation regardless of the initiating pathways (4). Therefore, pharmacological inhibition of AP could effectively inhibit complement activation and may be therapeutically beneficial in autoimmune disease.

The soluble regulator factor H (FH), composed of 20 short consensus repeats (SCRs), promotes the decay of C3 convertase in AP and downregulates the AP amplification. FH is considered to be the most important regulatory protein in AP thus holding great potential as a therapeutic intervention (5). Complement receptor of the immunoglobulin superfamily (CRIg) is exclusively expressed on tissue-resident macrophages and specifically binds to C3b/iC3b/C3c to inhibit AP and mediate opsonophagocytosis (6, 7). Based on their functions, a C3b/iC3b-targeted complement inhibitor, termed CRIg/FH, is developed by linking the extracellular functional domains of CRIg with the inhibitory domain of FH SCR1-5 (8). In previous studies, the therapeutic role of CRIg/FH has been verified in several inflammatory disorder models, including paroxysmal nocturnal hemoglobinuria (PNH) (8), mesangioproliferative glomerulonephritis (8), renal ischemia reperfusion injury (9), and lupus nephritis (10).

Myasthenia gravis (MG) is an autoimmune disease caused by antibodies targeting proteins expressed at the postsynaptic membrane of neuromuscular junction (NMJ). Approximately 85% of patients with generalized MG have detectable anti-acetylcholine receptor (AChR) autoantibodies. Despite current standard-of-care treatment, there is still a high degree of disease burden for many patients, suggesting a continued need for better treatment options (11).

AChR autoantibodies are predominantly IgG1 and IgG3, two subclasses that effectively activate complement (12). In MG, the antibodies bind and activate the complement cascade at the NMJ, resulting in the destruction of the postsynaptic membrane of skeletal muscle (13). Hence, the inhibition or modulation of complement is now recognized as a promising therapeutic strategy for MG. To date, three clinical trials of complement inhibition have been completed in MG. Eculizumab, a humanized monoclonal antibody that is directed toward the complement protein C5, has been approved by FDA for refractory generalized AChR-Ab–positive MG treatment after validation in phase 2 and phase 3 trials (14, 15). Another phase 2 clinical trial has demonstrated the efficacy and safety of zilucoplan, a small peptide C5 inhibitor, in a broader population of patients with moderate to severe generalized MG than those enrolled in the eculizumab studies (16). We hypothesized that CRIg/FH may also be beneficial to treat MG.

In this study, we examined the ability of CRIg/FH to inhibit complement activation induced by MG patient serum IgG *in vitro*, evaluated the *in vivo* therapeutic effect of CRIg/FH in both passive transfer myasthenia gravis (PTMG) and active experimental autoimmune myasthenia gravis (EAMG) models, and investigated possible humoral and cellular mechanisms involved in this effect.

## Methods

### Patient Samples

All MG patients were recruited at Huashan Hospital, Fudan University. Ten immunotherapy-naïve generalized MG patients who fulfilled the diagnostic standard (17) with confirmed relatively high titers of AChR-Ab (**Supplementary Table 1**) and ten sex- and age-matched healthy controls were included in this study. The study protocol was approved by the ethics committee of Huashan Hospital, Fudan University. Peripheral blood samples were obtained after informed consent.

### Animals

For PTMG and active EAMG studies, female Lewis rats 6–8 weeks of age (weighting 140–160 g) were purchased from the Vital River Laboratory Animal Co. Ltd. (Beijing, China) and housed in ventilated grommet cages (4 animals per cage) in the animal facility of the Institute in a controlled environment (12-h light/dark cycles; 23°C; specific pathogen free) with standard rat chow and water *ad libitum*. Soft food and water gels were placed on the bottom of the cages when an animal showed signs of weakness to ensure the animals were adequately fed and hydrated (18, 19). The studies were conducted following a protocol approved by the Administrative Panel on Laboratory Animal Care of Fudan University.

### Purification of IgG

Total IgG was purified from sera of MG patients and healthy control using a Melon gel IgG purification kit (Thermo Fisher Scientific, Waltham, MA) and was concentrated using Amicon Ultra centrifugal filter units (Millipore, Billerica, MA) according to the manufacturer’s instructions.

### Complement-Dependent Cytotoxicity

AChR-expressing TE671 cells (American Type Culture Collection, ATCC) were plated onto 96-well microplates at 50,000 cells/well and cultured in DMEM (Gibco BRL, Grand Island, NY) supplemented with 10% heat-inactivated fetal bovine serum (FBS, Gibco, Grand Island, NY), 100 units/ml penicillin, and 100 μg/ml streptomycin (Gibco) at 37°C/5% CO_2_ for 24 h. Cells were washed with complement-dependent cytotoxicity (CDC) buffer-Hanks’ buffered saline solution 10 mM HEPES, 1 mM sodium pyruvate, and 10 mg/ml Gentamicin (Invitrogen, Carlsbad, CA), and incubated at 37°C for 60 min with various concentrations of MG-IgG in CDC buffer in the presence or absence of CRIg/FH (60 μg/ml). Pooled normal human complement serum (10%, Innovative Research, Novi, MI) was used as a source of complement and incubated with TE671 cells in a final volume of 50 μl for 30 min at 37°C. Following MG-IgG treatment, TE671 cells were washed in CDC buffer, harvested, and then stained with 1 μl/ml fixable viability dye eFlour 780 (Invitrogen, Carlsbad, CA) for live/dead cell determination for 30 min at 4°C. The percentage of cell death was determined by acquiring flow cytometry data using Attune NxT Flow Cytometer (Thermo Fisher, Ramsey, MN). The percentage of fixable viability dye eFlour 780-positive cells (compared to total cells) was used to evaluate the degree of cell death.

### Immunocytochemistry Assay

TE671 cells were plated onto coverslips in 48-well plates and cultured at 37°C/5% CO_2_ for 24 h, and afterwards were incubated at 37°C for 60 min with MG-IgG (200 μg/ml) in the presence or absence of CRIg/FH (60 μg/ml). Pooled normal human complement serum (10%) was incubated with TE671 cells in blocking buffer (PBS containing 1% bovine serum albumin) for 30 min. Cells were then rinsed in PBS, fixed in 4% paraformaldehyde for 15 min. After fixation, cells were incubated for 2 h with rabbit anti-C5b-9n antibody (Abcam, Cambridge, MA), followed by mouse anti-rabbit IgG-conjugated Alexa Flour 488 and α-Bungarotoxin (BTX)-conjugated Alexa Flour 555 (Invitrogen, Carlsbad, CA) in 1% bovine serum albumin (BSA) for 30 min at room temperature. Following DAPI staining (Invitrogen, Carlsbad, CA) at a 10 μg/ml concentration, fluorescence was imaged on an Olympus fluorescence microscope. The quantitation of immunofluorescence images was performed with ImageJ.

### PTMG Model

PTMG was induced with anti-AChR mAb35 in female Lewis rats (18). The mAb35 (20 pmol/100 g) was administered i.p. 2 h after initial treatment with CRIg/FH (1 mg/100 g i.p). Rats were monitored for signs of clinical disease and weighed at 24 and 48 h post disease induction. Rats were sacrificed when weight loss exceeded 15% of starting weight; all remaining rats were sacrificed at 48 h. Clinical scores were as follows: 0, no weakness; 1, fatigable or weakness is only observed after exercise; 2, clinical signs of weakness present before exercise, hunched posture, or head down; 3, severe clinical signs of weakness: no ability to grip, hindlimb paralysis, respiratory distress/apnea, weight loss > 15%, immobility or moribund; 4, death (18). Diaphragms were harvested for histological analysis.

### Active EAMG Model

For active EAMG, rats were randomly divided into three groups (*n* = 8/group): the complete Freund’s adjuvant (CFA) control group, the active EAMG group, and the CRIg/FH treatment group. Rat AChR amino acid 97-116 peptide (R97-116, DGDFAIVKFTKVLLDYTGHI) was synthesized by Glbiochem (Shanghai, China) as described previously (20). For active EAMG induction, rats were immunized subcutaneously at the tail base with 50 μg R97-116 peptide emulsified in CFA mixed with 1 mg of *Mycobacterium tuberculosis* (strain H37RA; Difco, Detroit, MI) (21). Then, the rats were boosted on day 30 with the same dose of R97-116 in incomplete Freund’s adjuvant. The control group was injected with Freund’s adjuvant emulsified in PBS instead of the R97-116 peptide. Rats in the treatment group were given an intraperitoneal injection of CRIg/FH (1 mg/100 g) on day 29 and every 3 days thereafter. The remaining rats in CFA and active EAMG groups received intraperitoneal injection of PBS every 3 days. All animals were weighed at the beginning of the experiment. After the first immunization, body weights and clinical scores were assessed every other day in a blinded manner by two researchers at the same time until Day 62. Sera were collected for antibody and cytokine measurements on day 62 and stored at −80°C until use.

### ELISA for Serum Anti-R97-116 IgG Detection

Sandwich ELISA was performed to detect the anti-R97-116 antibodies. R97-116 (5 μg/ml) was coated onto 96-well plates (Corning, Corning, NY) at 4°C overnight. The plates were washed with PBS-T (PBS with 0.05% Tween 20) on the following day and then blocked for 2 h with 10% fetal calf serum at room temperature (RT). Rat serum samples diluted at 1:500 (100 μl/well) were added and incubated at RT for 2 h. After five washes, washed plates were incubated for 1 h with HRP-conjugated rabbit anti-rat IgG (1:2,000) at RT. Finally, TMB substrate solution was added, and the reaction was allowed to develop at RT in the dark. Plates were read at a wavelength of 450 nm and the results were expressed as OD value ± SD.

### Immunofluorescence for Serum Anti-AChR IgG Detection

The serum anti-AChR-IgG titer was measured with a live cell-based assay, using H9c2(2-1) cells lines (National Collection of Authenticated Cell Culture, Shanghai, China) expressing the rat muscle AChR (22). The H9c2(2-1) cells were seeded on the glass coverslips in 48-well plates for 24 h in DMEM (Gibco, Grand Island, NY) supplemented with 10% FBS (Gibco, Grand Island, NY) and 1% penicillin/streptomycin (Gibco, Grand Island, NY), then fixed by 4% paraformaldehyde for 15 min and washed three times with PBS. After blocking with 3% BSA for 1 h, serial dilutions of mAb35 or serum samples (in 0.5% BSA) were added and incubated at 4°C overnight. The rabbit anti-rat IgG conjugated with Alexa Flour 488 (1:1,000) and α-BTX conjugated with Alexa Flour 555 (1:500, Invitrogen, Carlsbad, CA) were incubated in 1% BSA for 60 min at room temperature. Following DAPI staining (Invitrogen, Carlsbad, CA), fluorescence was imaged on an Olympus fluorescence microscope. The quantitation of immunofluorescence images was performed with ImageJ (23). The mAb35 antibody was used at dilutions starting at 1:50 (0.174 mg/ml) and titrated in twofold serial dilutions. Fluorescence titration curve was obtained by plotting mean fluorescence intensity values against the log10 of the mAb35 concentration. Serum samples from active EAMG and CRIg/FH-treated rats were diluted twofold starting at 1:10 up to 1:1,280. Mean fluorescence intensities (serum samples diluted 1:80) were measured and used for calculation of anti-AChR antibody titers based on the titration curve.

### Immunofluorescence for NMJ Injury Detection

For analysis of MAC deposition at the NMJ, 10-μm transverse sections of rat frozen diaphragm muscle were sliced in both the control and experimental groups. Slides were fixed in cold acetone. After washing with PBS, the sections were blocked for 90 min with 4% BSA at room temperature and incubated overnight at 4°C with mouse anti-rat C5b-9n antibody (1:2,000, Santa Cruz, Heidelberg, Germany). After washing in PBS, Alexa Fluor488-conjugated anti-mouse IgG (diluted at 1:1,000, Abcam, San Francisco) and Alexa Fluor555-conjugated AChR-binding α-BTX (diluted at 1:2,000, Invitrogen, Carlsbad, CA) were incubated for 60 min. The sections were washed and then viewed with an Olympus fluorescence microscope.

### Flow Cytometry Analysis

Mononuclear cells (MNCs) were isolated from spleens of immunized rats on day 62 and prepared by grinding tissues through a cell strainer (Becton Dickenson, NJ) in lymphocyte separation fluid (Dakewe, Shanghai, China). MNCs were incubated with the following fluorochrome-conjugated monoclonal antibodies for 30 min at 4°C: anti-CD4 FITC and anti-CD25 PE (all from BD Bioscience, San Jose, CA). After the surface staining, cells were fixed and permeabilized with Intracellular Fixation and Permeabilization Buffer or Foxp3/Transcription Factor Buffer according to the manufacturer’s recommendations. After wash, cells were stained with anti-IFN-γ Alexa Flour 647A, anti-IL-17 PE, and anti-Foxp3 APC (eBioscience, San Diego, CA) to detect the intracellular or intranuclear markers. The expression of surface, intracellular, and intranuclear markers was analyzed by Attune NxT Flow Cytometer (Thermo Fisher, Ramsey, MN), and the results were analyzed using FlowJo software.

### Multi-Plex Analysis for Serum Cytokine

According to the previous studies in MG (24, 25), associated serum cytokines including IFN-γ, IL-2, IL-6, IL-10, and IL-17 were analyzed according to the manufacturer’s instructions, using a custom-made rat 5-plex kit. Briefly, following pre-wetting the filter plates, 25 μl of each sample/standard (in duplicate) was added to the bottom of the microplate wells. Then, 25 μl of bead suspension was added, and the plates were sealed with agitation overnight at 4°C. After the plates were washed twice, 25 μl of detection antibody was added; the plate was incubated for 60 min at room temperature. After incubation, 25 μl of streptavidin-PE was added to each well and incubated for 30 min at room temperature. The plate was again washed and 120 μl of reading buffer was added. The plate was read on a Bio-plex 200 analyzer.

### Treg Differentiation *In Vitro*

Active EAMG rats were induced with R97-116 peptide as described above and euthanized on day 45. Conventional CD4^+^CD25^-^ T cells were sorted from spleens of active EAMG rats by flow cytometry using BD FACS Aria II Cell sorter (BD Biosciences, San Jose, CA). The conventional T cells were cultured for 5 days in the presence of CRIg/FH with plate-bound anti-CD3 (2.5 μg/ml, Peprotech, Rocky Hill, NJ), anti-CD28(2.5 μg/ml, Peprotech), IL-2 (100U/ml, Peprotech), and TGF-β (1 ng/ml Sino Biological, Beijing, China) to induce iTreg differentiation. Foxp3^+^ cells were detected with flow cytometry.

### Statistical Analysis

Group assignments, data recording, and data analysis were blinded to the operator. Data distribution was tested *via* Kolmogorov test. For normal distributions, the difference between the groups was calculated by one-way or two-way ANOVA comparisons followed by multiple comparison test. Post-hoc tests were run only if *F* achieved *p* < 0.05 and there was no significant variance inhomogeneity. For non-normal distributions, the difference was calculated by Kruskal–Wallis test. When comparing two different groups, unpaired *t*-tests were used to determine statistical significance. Statistical analysis was performed using Prism 7 software (Graph Pad, La Jolla, CA) and a *p*-value < 0.05 was considered statistically significant.

## Results

### CRIg/FH Inhibits MG Patients’ Serum-Mediated Complement Activation on TE671 Cells

**Figure 1:**
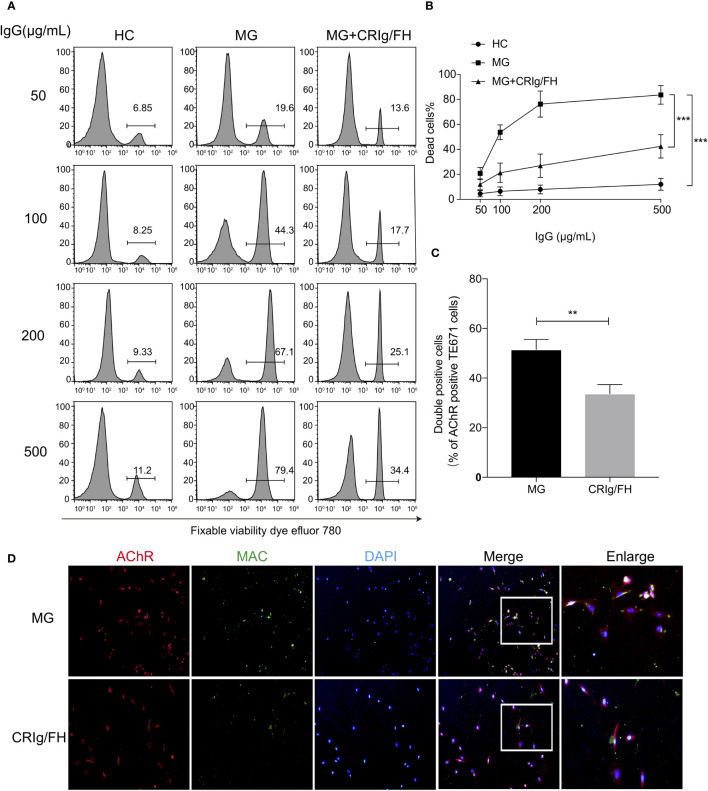
CRIg/FH inhibits MG IgG-mediated CDC and complement deposition on TE671 cells. **(A, B)** CRIg/FH protects TE671 cells from MG IgG-mediated CDC. TE671 cells were treated with various concentrations of MG-IgG in the presence or absence of CRIg/FH (60 μg/ml). Pooled normal human complement serum (10%) was used as a source of complement. The dead cells were stained by fixable viability dye eFluor 780 and detected by flow cytometry. The representative images in **(A)**, and the quantitative data as mean ± SD in **(B)**; *n* = 10/group, ***p < 0.0001; two-way ANOVA with Tukey’s multiple comparison test. **(C, D)** CRIg/FH prevents MAC deposition on TE671 cells. TE671 cells were incubated with MG patients derived IgG (200 μg/ml), normal human serum (10%) and CRIg/FH (60 μg/ml), and stained for MAC (anti-C5b-9n; green), AChR (identified by α-Bungarotoxin; red), and 4′,6-diamidino-2-phenylindole (DAPI; blue). Quantification of immunofluorescence images is shown in **(C)**. The percentage of double-stained cells (MAC and AChR) was quantified and plotted. Data are expressed as mean ± SEM; two-tailed *t*-test (**p < 0.01). Representative images are shown in **(D)**.

We first investigated the ability of CRIg/FH to suppress complement activation induced by the serum antibodies of MG patients. We collected the IgG fraction from the sera of 10 immunotherapy-naïve and AChR-Ab seropositive MG patients (**Supplementary Table 1**) to activate the complement classical pathway. The CDC effect was measured in the AChR-expressing TE671 cells by incubating with the purified serum IgG and using normal human sera as a complement source. We observed that the IgG from MG patients’ sera induced marked cell death in a dose-dependent manner compared to IgG from healthy control sera, while administration of CRIg/FH could potently suppress the CDC effect on TE671 cells (**Figures 1A, B**). We further explored the protective effect of CRIg/FH on TE671 cells by detecting MAC deposition on the cell membrane using Immunocytochemistry assay stained by C5b-9n antibody after incubation with the gMG patient-derived IgG and normal human sera. The result showed that CRIg/FH also remarkably reduced MAC assembly on TE671 cells (**Figures 1C, D**). Therefore, we demonstrated that CRIg/FH could effectively protect AChR-expressing TE671 cells from complement-mediated injury induced by gMG patient-derived IgG against AChR.

### CRIg/FH Prevents the Development of PTMG in Rats

**Figure 2:**
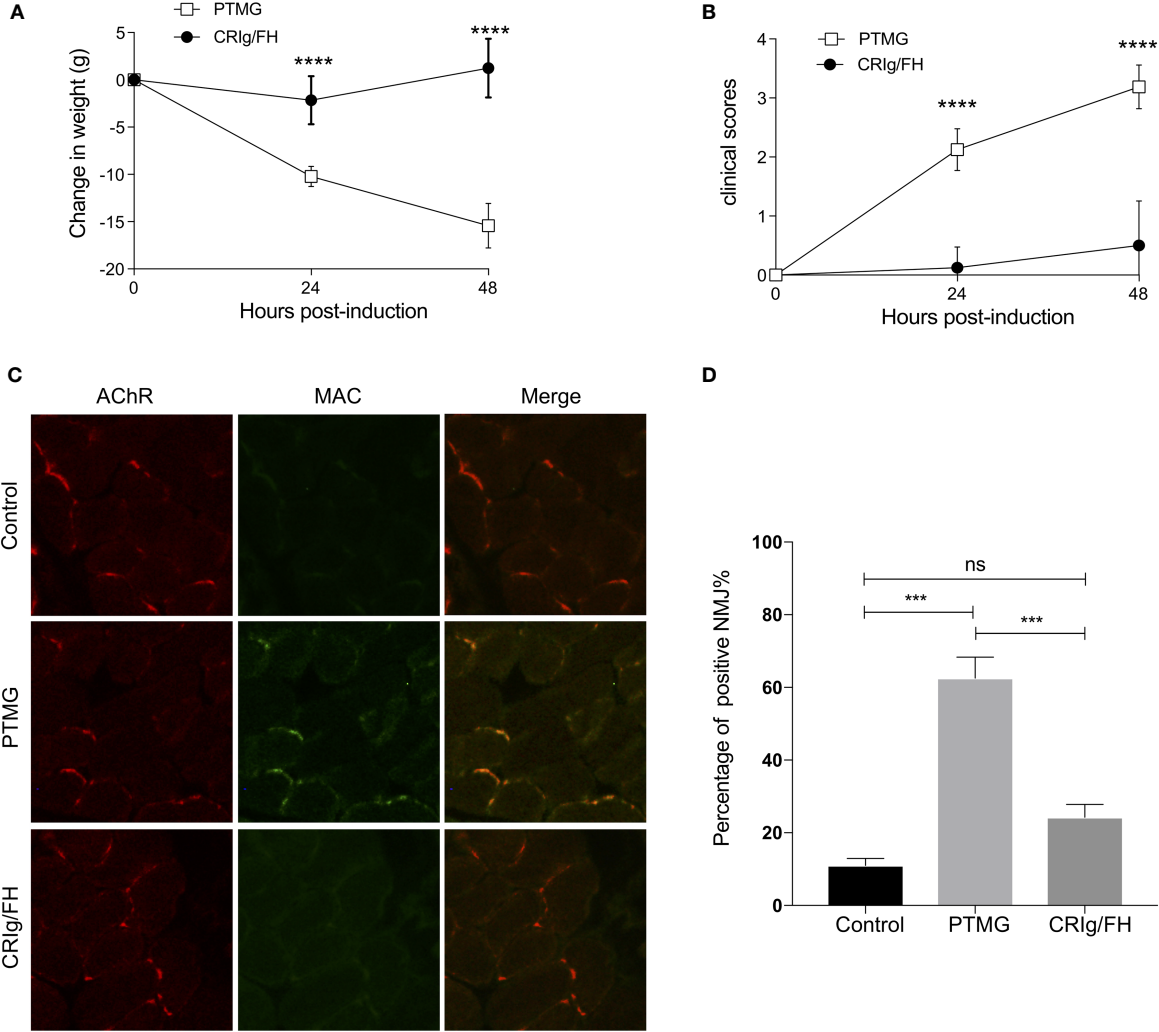
CRIg/FH prevents PTMG development in rats by restraining complement-mediated destruction of NMJs. **(A, B)** CRIg/FH ameliorates PTMG clinical outcomes potently. CRIg/FH treatment was administered 2 h before PTMG induction. The weight loss **(A)** and clinical score **(B)** were measured at 24 and 48 h after PTMG induction, respectively. Data are presented as mean ± SD; *n* = 6/group; ****p < 0.0001; two-way ANOVA with Sidak’s multiple comparison test. **(C, D)** CRIg/FH prevents MAC deposition on NMJs of PTMG rats. Diaphragm sections from control, PTMG, and CRIg/FH-treated rats were stained with anti-MAC (C5b-9n) antibody and detected with Alexa-488-labeled anti-rabbit IgG (green). Alexa-555-labeled bungarotoxin was used to identify AChR in NMJs (red). Minimum four sections with 5–15 NMJs from each diaphragm were quantified. Representative images are shown in **(C)** and quantitative data **(D)** are presented as the percentage of NMJs (%) with detected complement deposits (mean ± SEM). Comparisons of means were performed using one-way ANOVA with Tukey’s multiple comparison test (ns, not significant, ***p < 0.001). Results are representatives of three independent experiments.

The specific antibody against AChR may result in the NMJ injury promptly *via* complement activation (18); next, we evaluate the ability of CRIg/FH to prevent the induction of PTMG induced by passively transferring mAb35 into healthy rats. The results showed that mAb35 injection promptly induced the progressive and significant body weight loss (**Figure 2A**) and weakness represented by the clinical score (**Figure 2B**) at 24 and 48 h after injection, which may result from the complement attack determined by the extensive MAC deposition at the NMJ (**Figures 2C, D**). However, the pretreatment with CRIg/FH displayed a potent protective role in PTMG development. We observed no weight loss and little increase in clinical score after CRIg/FH pretreatment in PTMG animals (**Figures 2A, B**). The results of muscle pathological detection further confirmed that MAC deposition at the NMJ was near completely suppressed by the CRIg/FH pretreatment (**Figures 2C, D**). Thus, these findings demonstrated that CRIg/FH could prevent the development of PTMG by inhibiting complement activation.

### CRIg/FH Ameliorates Disease Severity of Active EAMG Rats and Reduces Autoantibody Titers

**Figure 3:**
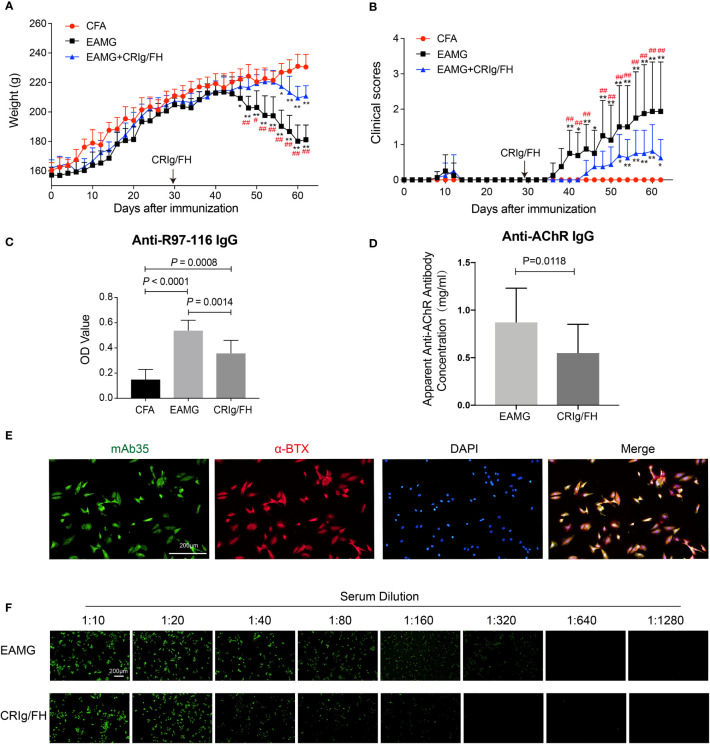
CRIg/FH treatment ameliorates active EAMG and reduces the serum concentration of anti-AChR antibody in rats. **(A, B)** CRIg/FH ameliorates active EAMG clinical outcomes significantly. CRIg/FH was injected into active EAMG rats every 3 days from the day before the second immunization (day 29). The rats were sacrificed at the 62nd day after the first immunization, and the rat sera were collected. The body weights **(A)** and clinical scores **(B)** were recorded every other day. Arrows indicate the days that CRIg/FH were started. **(C, D)** CRIg/FH reduces the serum levels of auto-antibodies. The serum antibody against AChR 97-116 peptide was measured by ELISA **(C)** and the apparent anti-AChR antibody concentration was determined by immunofluorescence with H9c2(2-1) cells expressing rat muscle AChR, corresponding to a concentration of mAb35, which gives an equivalent fluorescent signal on H9C2(2-1) cells **(D)**. Data are presented as mean ± SD **(A–D)**; *n* = 8/group; two-way ANOVA **(A, B)**, one-way ANOVA **(C)** with Tukey’s multiple comparison test or Student’s *t*-test **(D)**; *p < 0.05, **p < 0.01, significant differences compared with the CFA group at each indicated time point; ^#^ *p* < 0.05, ^##^ *p* < 0.01, differences between active EAMG and active EAMG+CRIg/FH groups at each time point. Results are representative of three independent experiments repeated with similar results. **(E)** Stable expression of rat AChR subunits in H9c2(2-1) cells. H9c2(2-1) cells were incubated with anti-AChR mAb35 (21.7 μg/ml) and stained for rabbit anti-rat IgG conjugated with Alexa Flour 488 (green), AChR (identified by α-Bungarotoxin; red), and 4′,6-diamidino-2-phenylindole (DAPI; blue). Scale bars, 200 μm. **(F)** Immunofluorescence assay endpoint titer results for serum samples against rat AChR subunits in H9c2(2-1) cell. H9c2(2-1) cells were incubated with serum samples at dilution titers from 1:10 up to 1:1,280 and stained for rabbit anti-rat IgG conjugated with Alexa Flour 488 (green). Scale bars, 200 μm.

We next evaluated the therapeutic potential of CRIg/FH in the active EAMG model rats immunized with AChR peptide (R97-116). Compared to the adjuvant control, immunization with AChR peptide caused significant body weight loss starting at day 46 through the end of the experiment (**Figure 3A**), and progressive exacerbation in clinical symptoms starting from day 40 until the end of the experiment (**Figure 3B**). In the CRIg/FH-treated group, we injected drug (1 mg/100 g, i.p.) every 3 days starting from day 29, and found a strong therapeutic effect determined by the measurement of body weight loss and clinical score. As shown in **Figure 3A**, the body weight of CRIg/FH-treated rats started to decrease on day 56, which was delayed, compared to the rats in the active EAMG group; moreover, the body weight loss was also significantly lower than the control active EAMG rats. In addition, the observation in clinical scores also showed similar results. The clinical scores started to significantly increase until day 52 in the CRIg/FH-treated group, in which the increase occurred 12 days later, and the score was significantly lower than the active EAMG group without CRIg/FH treatment (**Figure 3B**).

Considering the role of complement activation in B-cell development and autoantibody production *via* its components such as CR2 (26) and FH (27), we further detected the production of specific antibodies against R97-116 and AChR in these rats with sera collected on day 62. The levels of anti-R97-116 IgG were measured by ELISA and the titers of anti-AChR IgG were determined by immunofluorescence using the cell-based assay described above. We observed that the combined immunization with R97-116 peptide and CFA effectively induced the production of specific antibody against R97-116 peptide and AChR in active EAMG model rats, while CRIg/FH treatment could remarkably reduce the serum levels of both anti-R97-116 and anti-AChR IgG (**Figures 3C–F**). These results suggested a potential mechanism for CRIg/FH preventing active EAMG development *via* inhibition of specific antibody production. Together, we further demonstrate that CRIg/FH holds the therapeutic potential in the active EAMG model, by both regulating humoral response and directly inhibiting complement activation.

### CRIg/FH Reduces the Production of Pro-Inflammatory Cytokines in Active EAMG Rats

**Figure 4:**
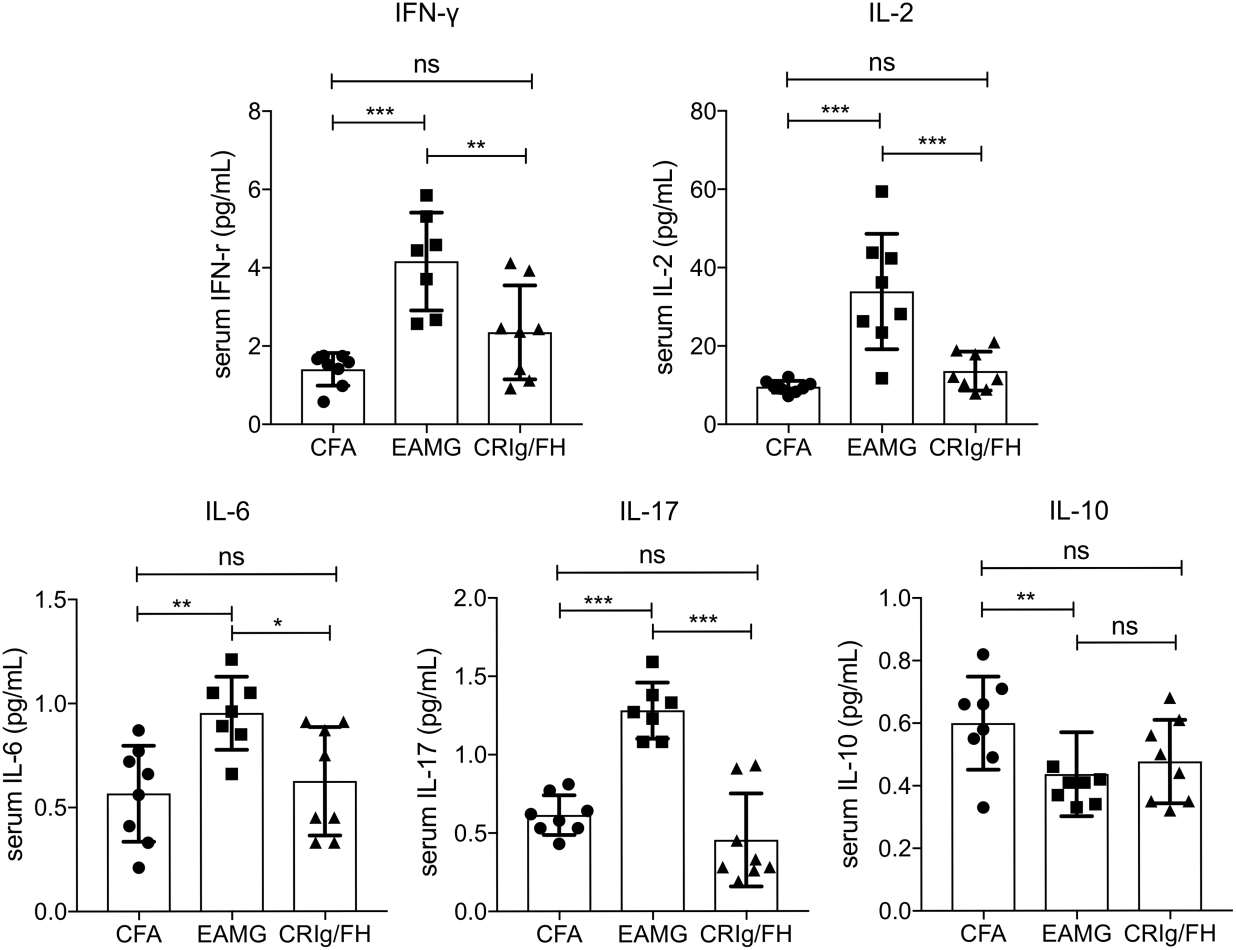
The effect of CRIg/FH treatment on cytokine production in active EAMG rats. Rat sera were collected to measure the concentrations of the cytokines of IFN-γ, IL-2, IL-6, IL-10, and IL-17. Data are presented as mean ± SD. Statistical significance was assessed by one-way ANOVA test with Tukey’s multiple comparison test; *n* = 7–8/group; ns, not significant, *p < 0.05, **p < 0.01, ***p < 0.001.

In inflammatory diseases, complement activation may induce the production of cytokines and chemokines through the regulation of the downstream cellular immunity (1, 28, 29). A bunch of pro-inflammatory cytokines has been reported to be upregulated in active EAMG and MG, including IFN-γ, IL-2, IL-6, and IL-17 (30–33). Thus, we measured the serum concentration of IFN-γ, IL-2, IL-6, IL-17, and IL-10 to further understand the protective mechanisms of CRIg/FH in active EAMG rats. After immunizing with AChR peptide R97-116 in CFA, the active EAMG rats exhibited a significant elevation of IFN-γ, IL-2, IL-6, and IL-17 (pro-inflammatory), and decrease of IL-10 (anti-inflammatory) compared to the CFA alone control treatment (**Figure 4**). However, CRIg/FH treatment remarkably reduced the serum levels of IFN-γ, IL-2, IL-6, and IL-17 in treated rats when compared to that in untreated active EAMG rats. More importantly, CRIg/FH treatment strongly reduced the serum levels of these cytokines to that of the adjuvant control (**Figure 4**). In addition, CRIg/FH treatment failed to alter the serum level of IL-10 in active EAMG rats (**Figure 4**). Therefore, we determined that CRIg/FH could potently downregulate the production of multiple pro-inflammatory cytokines in active EAMG rats.

### CRIg/FH Promotes Regulatory T Cell Differentiation

**Figure 5:**
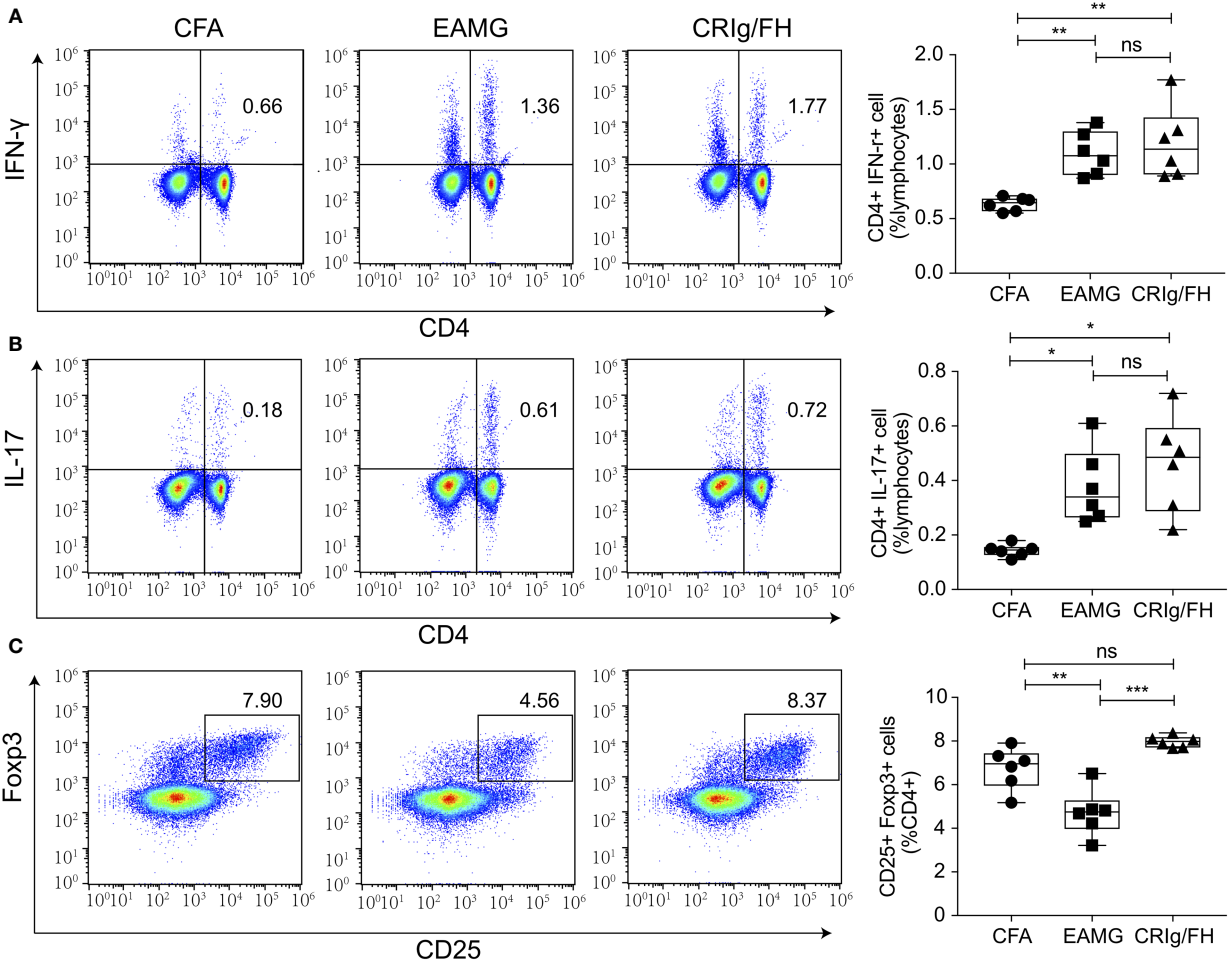
CRIg/FH treatment upregulates Treg subset in active EAMG rats. **(A–C)** The levels of Th1 **(A)**, Th17 **(B)**, and Treg **(C)** in the spleen. Expression levels of Th1 (CD4^+^IFN-γ^+^), Th17 (CD4^+^IL-17^+^), and Treg (CD4^+^CD25^+^Foxp3^+^) cells were determined by flow cytometry. Data are expressed as mean ± SD; *n* = 6/group; ns, not significant, *p < 0.05, **p < 0.01, ***p < 0.001; one-way ANOVA test with Tukey’s multiple comparison test.

Treg cells play a critical role in the prevention of MG by suppressing the effector CD4^+^ T cell subsets and maintaining immune homeostasis and self-tolerance (34, 35). The blockage of complement C3aR and C5aR may promote TGF-β1-induced Treg differentiation (36), and both of CRIg and FH have been reported to suppress T-cell activation (37–39). Therefore, we examined the effect of CRIg/FH treatment on the development of T helper and Treg cells in active EAMG rats. Significant increase of Th1 (CD4^+^IFN-γ^+^) and Th17 (CD4^+^Th17^+^) and decrease of Treg (CD4^+^CD25^+^ Foxp3^+^) cells in the spleen of active EAMG rats were observed compared to the CFA alone treatment rats (**Figure 5**), indicating the involvement of various CD4^+^ T subset cells in active EAMG development. However, CRIg/FH only upregulated Treg subsets while displaying no significant effect on the Th1 and Th17 cells (**Figure 5**).

**Figure 6:**
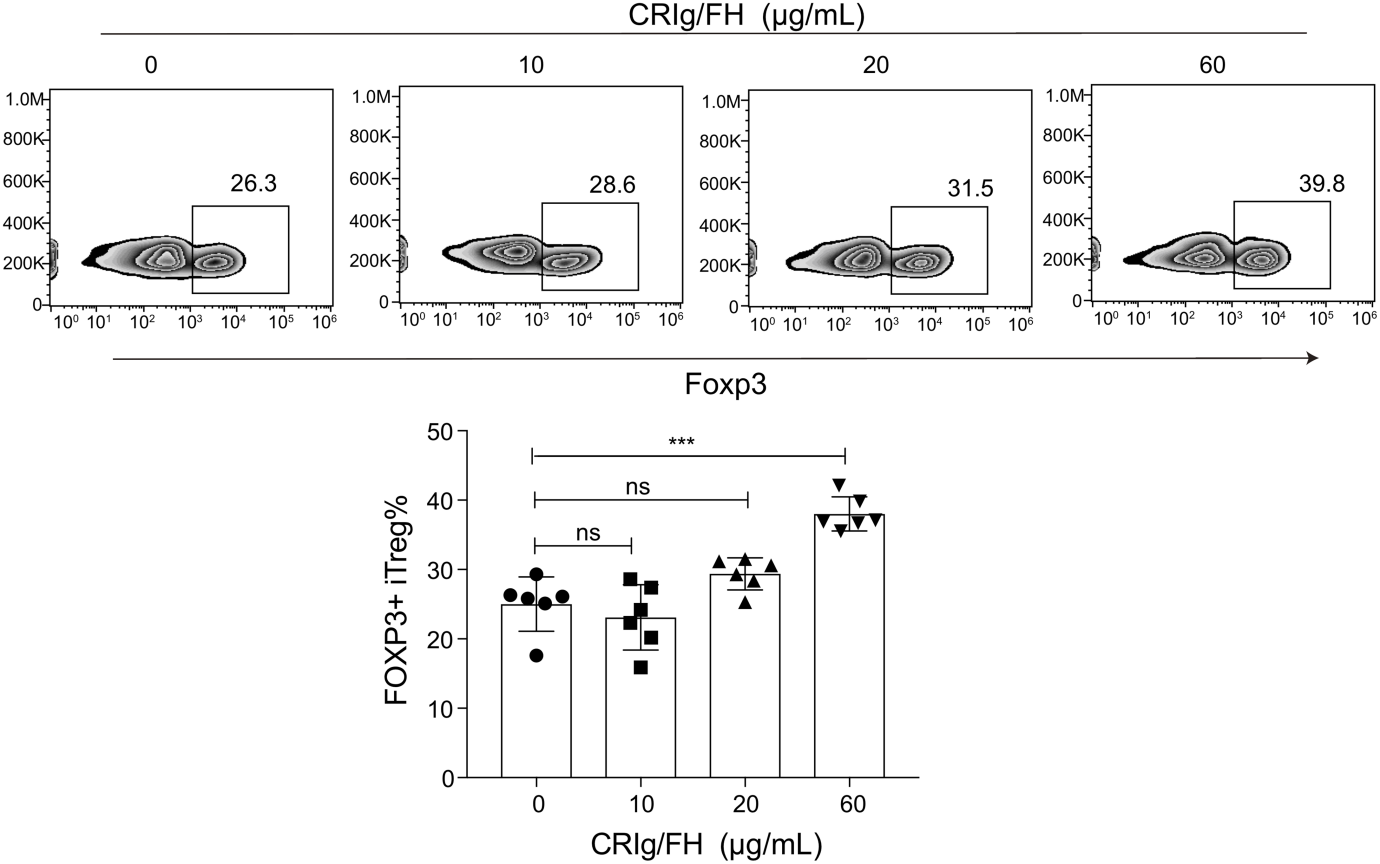
CRIg/FH promotes Treg differentiation *in vitro*. CD4^+^CD25^−^ conventional T cells were stimulated with immobilized anti-CD3, anti-CD28 antibodies, IL-2, and TGF-β in the absence or presence of the indicated concentrations of CRIg/FH. Foxp3 expression was assessed by flow cytometry on day 5 after stimulation. Representative images and quantitative data showed the generation of iTreg cells under various concentrations of CRIg/FH treatment. Results are expressed as means ± SD, *n* = 6/group, and ns, not significant, ***p < 0.001 determined by one-way ANOVA followed by Dunnett’s multiple comparison test.

We further assessed whether CRIg/FH could promote Treg differentiation induced by TGF-β *in vitro*. The isolated CD4^+^CD25^-^ conventional T cells from the spleens of Lewis rats were cultured in the presence of anti-CD3/CD28, IL-2 and TGF-β to induce iTreg differentiation. The additional CRIg/FH treatment may significantly promote iTreg differentiation at 60 μg/ml (**Figure 6**). Therefore, these findings revealed another mechanism by which CRIg/FH may protect from MG *via* the promotion of Treg differentiation.

## Discussion

In the present study, we explored the effect of a targeted complement inhibitor, CRIg/FH, on MG. Our results demonstrated that CRIg/FH inhibited CDC and complement deposition produced by IgG from AChR-Ab positive MG patients *in vitro*. Then, we evaluated the *in vivo* effect of CRIg/FH in the passive and active rat model of MG. The PTMG model did not recapitulate the complexities of the breakdown of self-tolerance in human MG but reproduced the common pathway of antibody-induced, complement-mediated destruction of NMJ. Administration of CRIg/FH prevented the development of PTMG rats and reduced MAC deposition at the NMJ. Acitve EAMG model induced by R97-116 peptide immunization leads to a loss of self-tolerance and auto-antibody production, as occurs in human MG. CRIg/FH ameliorated the disease severity of active EAMG rats, accompanied by the reduced anti-AChR autoantibodies, decreased serum levels of IFN-γ, IL-2, IL-6, and IL-17, and increased Treg differentiation.

Numerous studies from murine disease models and human patients have suggested that the complement hyperactivation plays a principal role in MG (40). In contrast to the current immunosuppressive therapies (e.g., corticosteroids and other immunosuppressants), which nonspecifically suppress the immune response, the complement inhibition represents a potential strategy toward suppressing the primary effector mechanism of the destruction of the post-synaptic membrane of the NMJ in MG (41). A large number of anti-complement drugs are in development, providing tools for blocking the whole complement activity, the specific activation pathways, or the active fragments. In addition to the clinically approved C5 antibody, other approaches including decay accelerating factor (DAF, CD55)-containing targeted fusion protein, siRNA specific against C2, and antibody against C6 have been shown effective to reduce the severity of active EAMG (42–44).

CRIg/FH is a recombinant protein consisting of the C3b/iC3b-binding domain of CRIg and the inhibitory domain of FH (SCR1-5). It can inhibit complement activation *via* classical and alternative pathways specifically at the sites of complement activation (8), which, in the case of AChR antibody-induced MG, is the post-synaptic membrane of NMJ. CRIg could target C3b/iC3b, thus leading to the inhibition of complement activation mainly *via* an alternative pathway (6, 45). FH promotes dissociation of C3 convertase and, together with factor I, mediates proteolytic inactivation of C3b (46). Therefore, through its target C3b/iC3b that already covalently inserts cell membrane, CRIg/FH could deposit specifically at the complement activation sites with a long duration. Hence, it may provide long-lasting local cell surface and tissue-based inhibition of complement-induced damage (8). In our study, a single dose of CRIg/FH was sufficient to prevent the development of hind-limb paralysis and reduced C5b-9 deposition on NMJ in PTMG rats, suggesting that it could target to and be retained at the NMJ site for a sufficient time.

Apart from their essential roles in complement regulation, other immune modulatory effects have been suggested for CRIg and FH. CRIg has been identified to serve as a co-inhibitory molecule that suppressed CD4^+^ Th cell activation and cytokine production (38, 39). FH has been suggested to induce an anti-inflammatory state in monocyte-derived dendritic cell (37) and modulate cell activation through binding to specific receptors in the host cell (47). In the current study, we found that CRIg/FH treatment reduced the serum levels of anti-R97-116 and anti-AChR antibodies. Our previous study found similar results in a murine lupus nephritis model, in which CRIg/FH also reduced the titer of auto-antibody against ds-DNA (10). In addition, Jindrich Soltys et al. demonstrated a decrease in complement fixing anti-AChR IgG1 antibody in EAMG rats treated with rEV576, a C5 complement inhibitor (48). This reduction of auto-antibody may result from the complement inhibition. Two complement receptors, CR1 and CR2, whose ligands C3b/iC3b are also the targets of CRIg/FH, play important roles in the generation, quality, and maintenance of serum autoantibodies (49). Furthermore, we also found that CRIg/FH suppressed the production of inflammatory cytokines IFN-γ, IL-2, IL-6, and IL-17 while promoting the differentiation of Treg in active EAMG rats, indicating that CRIg/FH had an extra impact on adaptive immunity. Given the dual function of CRIg/FH in inhibiting complement activation and regulating humoral immune response, it may be effective not only in patients with acute disease flares but also for maintaining treatment of chronic MG.

It should be noted that inconsistent changes in serum cytokines and in corresponding cytokine-producing Th cell subsets were found in our study. This may due to the fact that the cytokines involved in the present study could be generated by different immune cells, and one type of immune cell could also give rise to a variety of cytokines. For example, IL-17 could be produced by Th17 cell, Type 17 CD8+ T cell, and γδT cell (50). Therefore, the complexity of immune cellular network might result in the inconsistency observed in our results. In addition, although we observed that CRIg/FH upregulated Treg cells *in vivo* while promoting Treg differentiation *in vitro*, we cannot conclude that CRIg/FH affects Treg differentiation directly *in vivo*. The possibility that the upregulation of Treg cells was secondary to the inhibition of autoimmune response under *in vivo* conditions cannot be excluded, since relative deficiency of Treg cells was seen in autoimmune response to AChR and pharmacokinetics are very different between *in vitro* and *in vivo*. Further studies are still needed to explore the mechanisms by which CRIg/FH downregulates pro-inflammatory mediators and promotes the differentiation of Treg cells.

In conclusion, our results demonstrated that the targeted complement inhibitor CRIg/FH may hold great potential for MG treatment, in which the therapeutic effect results not only from the direct complement inhibition but also from the modulation of adaptive immune responses. Thus, these pharmacodynamic results provide a rationale for testing CRIg/FH in MG patients in the future.

## Data Availability Statement

The raw data supporting the conclusions of this article will be made available by the authors, without undue reservation.

## Ethics Statement

The studies involving human participants were reviewed and approved by Huashan Hospital, Fudan University. The patients/participants provided their written informed consent to participate in this study. The animal study was reviewed and approved by Fudan University.

## Author Contributions

JS, RZ, CY, PPD, and LL performed the research, analyzed the data, and wrote the manuscript. SL and JX contributed to the conception and design of the study. WH and CZ participated in the design of the study and helped to draft the manuscript. All authors contributed to the article and approved the submitted version.

## Funding

This work was supported by financial grants from the National Natural Science Foundation of China (No. 82071410 and 81870988 to CZ, 82001335 to JS, and 81790254, 91629301, 81872354 and 82121004 to WH), Shanghai Municipal Science and Technology Major Project (No. 2018SHZDZX01), and ZJLab.

## Conflict of Interest

The authors declare that the research was conducted in the absence of any commercial or financial relationships that could be construed as a potential conflict of interest.

## Publisher’s Note

All claims expressed in this article are solely those of the authors and do not necessarily represent those of their affiliated organizations, or those of the publisher, the editors and the reviewers. Any product that may be evaluated in this article, or claim that may be made by its manufacturer, is not guaranteed or endorsed by the publisher.

## Supplementary Material

Supplementary Table 1 Clinical characteristics of MG patients.
