# Supplementary material for: A Targeted Complement Inhibitor CRIg/FH Protects Against Experimental Autoimmune Myasthenia Gravis in Rats via Immune Modulation
Source: Front Immunol. 2022 Jan 26;13:746068. doi: 10.3389/fimmu.2022.746068 (PMC8825366; doi:10.3389/fimmu.2022.746068)
Supplement: Supplementary Table 1 — Clinical characteristics of MG patients. [file Table_1.docx]

**Supplementary Table 1**  Clinical characteristics of MG patients

| Specimen ID | Age (y) | Male/Female (M/F) | MGFA classification | QMGs | AChR-Ab (nmol/L) |
| --- | --- | --- | --- | --- | --- |
| 1 | 58 | M | IIIb | 27 | 16.89 |
| 2 | 49 | M | IIa | 9 | 13.59 |
| 3 | 32 | F | IIa | 13 | 13.11 |
| 4 | 45 | F | IIa | 12 | 15.63 |
| 5 | 46 | F | IIa | 6 | 14.87 |
| 6 | 50 | M | IIb | 9 | 14.44 |
| 7 | 21 | M | IIa | 12 | 15.78 |
| 8 | 67 | F | IIb | 9 | 22.04 |
| 9 | 33 | F | IIIa | 21 | 14.76 |
| 10 | 29 | M | IIa | 9 | 14.64 |
